# Quality of life in small-scaled homelike nursing homes: an 8-month controlled trial

**DOI:** 10.1186/s12955-018-0853-7

**Published:** 2018-02-27

**Authors:** Jeroen S. Kok, Marjan M. A. Nielen, Erik J. A. Scherder

**Affiliations:** 1 Lentis|Dignis, Mental Health Care Institute, PO Box 128, 9470 AC Zuidlaren, The Netherlands; 2 ZINN, Mental Health Care Institute, PO Box 51, 9750 AB Haren, The Netherlands; 3 Department of Clinical Neuropsychology, VU University Amsterdam, van der Boechorststraat 1, 1081 BT Amsterdam, The Netherlands

**Keywords:** Dementia, Long term care, Nursing home, Quality of life

## Abstract

**Background:**

Quality of life is a clinical highly relevant outcome for residents with dementia. The question arises whether small scaled homelike facilities are associated with better quality of life than regular larger scale nursing homes do.

**Methods:**

A sample of 145 residents living in a large scale care facility were followed over 8 months. Half of the sample (*N* = 77) subsequently moved to a small scaled facility. Quality of life aspects were measured with the QUALIDEM and GIP before and after relocation.

**Results:**

We found a significant Group x Time interaction on measures of anxiety meaning that residents who moved to small scale units became less anxious than residents who stayed on the regular care large-scale units. No significant differences were found on other aspects of quality of life.

**Conclusions:**

This study demonstrates that residents who move from a large scale facility to a small scale environment can improve an aspect of quality of life by showing a reduction in anxiety.

**Trial registration:**

Current Controlled Trials ISRCTN11151241. registration date: 21–06-2017. Retrospectively registered.

## Background

Improving the Quality of Life (QOL) in residents with dementia would be a clinical most relevant treatment outcome, since there is no cure for this progressive syndrome. QOL is a multidimensional concept [1–3]. One of the practical difficulties in assessing QOL in dementia is that the cognitive functions that are needed to respond adequately to the interview questions usually deteriorate with the progression of the syndrome [4]. Therefore, QOL is usually measured using more indirect observational measures [5–7]. One concern with respect to the validity of these observation measurements is that their assessment significantly depends on the clinical experience of the investigator [8, 9].

There are significant relationships between proxy based QOL measurements such as positive affect on the one hand and severity of neuropsychiatric symptoms such as agitation and depression on the other hand [10]. Severity of depressive symptoms shows the strongest relationship with QOL [11, 12].

Special Care Units (SCU) for residents with dementia can provide an increase of QOL [13–15]. In the Netherlands most persons with severe dementia reside in two different types of SCUs; regular SCUs and Small Scaled Homelike SCUs. There are also nursing homes for elderly persons with less severe dementia or somatic disabilities. In regular SCUs, residents are nursed in groups of 20–30 persons a ward with shared bedrooms [16, 17] whereas small scaled homelike SCUs provide care for groups of 7–8 persons with individual bedrooms [18]. In small scaled homelike facilities the staff are trained in person-centered care [19] and the residents participate in meaningful household activities such as cleaning, preparing meals and folding up the laundry. The daily environment encompasses a kitchen, garden and other homelike elements [18]. In regular SCUs household activities are coordinated centrally with little or no cooperation of the residents [20].

In the Netherlands it is generally assumed that small scaled homelike SCUs are more beneficial concerning social support, safety, familiarity and experienced congeniality for residents with dementia compared to regular SCUs [21]. Consequently, there has been a tendency over the last decade to convert from regular to small scaled homelike SCUs [21].

Research shows different effects in the perception of nursing personnel and family. It has been shown that nursing personnel in small-scale units experience less workload, more autonomy and more social support from their colleagues [21] or no difference in job satisfaction and motivation [22]. Family members experience less burden and report more satisfaction with the nursing staff [22].

In this study we examined the possible beneficial effects on quality of life of older people with moderate to severe dementia who moved from a large SCU to a small scaled homelike SCU. Based on earlier research [23, 24], our hypothesis was that residents in a small scaled homelike SCU experience more quality of life than residents living in regular care.

## Methods

### Study design

The study is an 8-month longitudinal non-randomized experimental study in which people stayed in an intervention and control group.

### Settings

Residents with advanced dementia mostly live in specially designed homelike SCUs in the Netherlands. Residents live in wards with 7–8 residents instead of living in regular SCUs with 20–30 residents in one ward. Most small scaled facilities are located within a larger care facility.

The participating SCUs we selected were comparable at the start of the study in terms of number of residents, type and number of staff members, facility size and geriatric treatment. The residents lived in non-profit collective nursing homes for residents with dementia in the Northern part of the Netherlands. In one nursing home, residents moved to a small scaled homelike facility because the building did no longer meet the current health care standard (experimental group). The control group was selected from within the same institute in order to ensure comparability between both groups.

### Procedure

At the start of the study, all residents stayed in two regular SCUs, with 20–30 residents per ward, of one mental health care institute in the Netherlands. One group, which was connoted as the intervention group, moved after 2 months to a small scaled homelike SCU, with 7–8 residents per ward, situated in a large building. The entire nursing staff of the intervention group received a training focused on the new accommodation. This 9-h training was focused on person-centered care [25] for residents with dementia. This training was performed by external and internal trainers and was part of the organizational transfer of the residents.

**Fig. 1:**
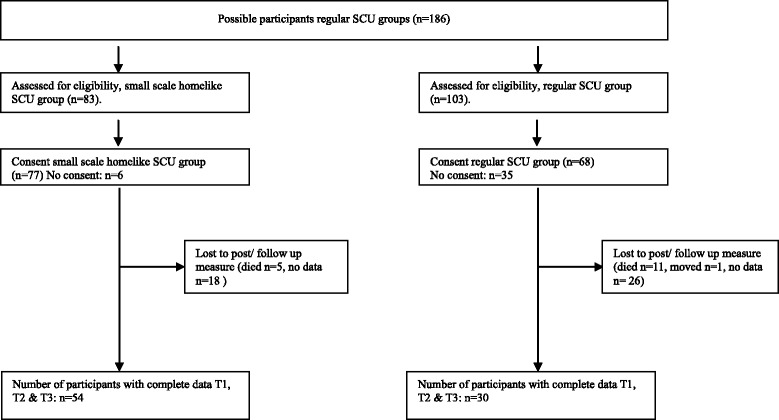
Flow chart, inclusion of patients

Residents in the control group remained in their large-scale living environment and received care as usual with no special training for the personnel. The resident – staff ratio and allocation to residents stayed the same for both conditions. After 3 and 6 months, the assessment procedure was repeated in both groups. The intervention group was located in a rural village, the control group in a small city. For a flowchart of resident inclusion, see Fig. 1.

Different proxy measurements, a questionnaire and information from the medical records were used to collect the data. Part of the data was collected directly from all residents by specially trained research assistants (global cognitive functioning and mood). Other data were collected by trained primary nurses (QOL and neuropsychiatry). The research assistants and primary nurses were trained by a senior psychologist during a specially organized training session.

The observation lists of a single resident were filled in by two nurses in order to improve the inter-rater reliability. The research leader was available and could be consulted during the moments of data collection. The data collection was performed within the different care facilities and therefore not blinded.

### Ethical issues

The study was approved by the Ethical Committee of the department of Psychology of the University of Groningen, the Netherlands (no. PPO008093), registered 3 June 2009. The legal representatives of the residents provided written informed consent and the residents verbal consent. In case the resident expressed either verbally or non-verbally the wish to end the examination, the examination was directly terminated.

### Materials

#### Global cognitive functioning

The Standardized Mini Mental State Examination (SMMSE) [26] was used to measure global level of cognitive functioning. This screening instrument contains items measuring long and short term memory, orientation, constructional ability, language and the ability to follow up commands. A higher score stands for better cognitive functioning (the maximum score is 30).

#### Mood

Besides the above proxy rating of symptoms of depression, the Geriatric Depression Scale (GDS) [27–29] was used to measure symptoms of depression directly from the resident with dementia. Questions were presented verbally to the resident and contains a 15-point scale. A score of 5 points or higher is considered as a cut off for the presence of depression [27].

#### Quality of life

We used the QUALIDEM as an estimate for quality of life [30–32]. This observation instrument is specially designed to assess the quality of life of residents with dementia in SCUs. The validity and reliability is sufficient [31]. The QUALIDEM contains nine subscales of behavior with 40 items rating; care relationships (7 items), positive affect (6), negative affect (3), restless tense behavior (3), positive self-image (3), social relations (6), social isolation (3), feeling at home (4) and having something to do (2). All nine dimensions were used for this study. Each scale contains a four-point scale (never, seldom, sometimes, often). A higher score indicates a higher quality of life.

#### Neuropsychiatry

Relevant neuropsychiatric symptoms were collected with subscales of the Behavioral Observation Scale for Intramural Geriatric Psychiatry (Gedragsobservatieschaal voor Intramurale Psychogeriatrie (GIP)) [33]. This observation list is specially designed for observation of residents with dementia in SCUs and contains 14 subscales. We used the 6 dimensions that we believed to be most relevant to a study of quality of life; namely Not social behavior (maximum score 24), Apathy (maximum score 18), Insubordinate behavior (maximum score 15), Suspicious behavior (maximum score 21), Depressive behavior (maximum score 18) and Anxious behavior (maximum score 18). Each scale contains a four-point scale (minimum score of 1, maximum score of 4). A higher score indicates a worse outcome.

### Data analysis

Data were analyzed using SPSS version 24. Descriptive analyses were used to present the characteristics of the residents. Possible differences between the intervention and control group at baseline were assessed by independent *t*-tests and Chi-squared tests.

We applied univariate analyses of covariance to analyze the dependent variables QUALIDEM and GIP to explore possible differences in quality of life over time (three moments of measurement) between the two resident groups: two months before intervention (T0), 3 months after intervention (T1) and 6 months after intervention (T2). The baseline-score was used as covariate and the post or follow-up scores as dependent variable. The between subjects factors were the type of SCU (large vs small) and the individual resident.

Level of significance was set at *p* < .05. Effect sizes (eta squared, 95% CI) between .01–.05 was considered small, between .06–.13 moderate and .14 and higher large [34].

## Results

### Resident characteristics

At baseline, we included a total number of 145 residents living in two large nursing facilities. They were all residents of SCUs in a moderate to severe stage of dementia as reported in their medical records. To recruit residents, two nursing homes were invited to participate (*N* = 186). A total number of 145 legal representatives of participants provided written and verbal informed consent to the study, 41 representatives did not respond. Due to missing data (*N* = 44), diseased (*N* = 16) and moving to another nursing home (*N* = 1), 84 residents could be included eventually. Part of the data of this sample has been also been described in other studies [35, 36].

### Baseline characteristics

Resident characteristics at baseline are presented in Table 1. We found no significant differences between the large scale unit group and the small-scale unit group concerning age, gender, level of education, global cognitive functioning and mood. Both groups consisted of residents that were over 80 years of age, mostly female who generally had followed elementary levels of education (i.e. primary school and no further education) [37]. Type of dementia was registered according to the information in the medical record (Table 1). Level of global cognitive functioning (as estimated with the standardized MMSE) could be categorized as severely impaired. In both groups, mean scores on the GDS indicated that in either group, residents showed depression scores below cut off (see Table 2).

**Table 1:** Resident characteristics at baseline

Dementia type	Small scale homelike SCU group *N (%)*	Regular SCU group *N (%)*
Dementia nos	18 (23)	26 (38)
Alzheimers’ dementia	24 (31)	13 (19)
Mixed dementia	6 (8)	11 (16)
Vascular dementia	5 (7)	8 (12)
Lewy body dementia	1 (1)	1 (2)
Frontotemporal dementia	0 (0)	4 (6)
Other^a^	4 (5)	1 (2)

^a^Parkinson dementia, alcohol dementia, Korsakov, semantic dementia, corticobasal degeneration

**Table 2:** Type of dementia of the residents

	Small scaled homelike group	Regular care group	Test statistic*
	M(SD)^a^	M(SD)^a^	
Sample size (*n*)	77	68	
Age	83.4 (6.1)	82.8 (7.6)	*.611* ^*1*^
Gender			.671^1^
Male	24 (31%)	19 (28%)	.674^1^
Female	53 (69%)	49 (72%)	
Education^b^	3.3 (1.4)	3.3 (1.4)	*.901* ^*2*^
Global cognitive function^c^	8.7 (6.5)	8.4 (6.5)	.802^1^
Mood^d^	1.3 (1.2)	1.0 (0.8)	.187^1^

^a^unless indicated otherwise

^b^conform Verhage [37], Low = category 1 + 2 + 3^b^, Middle = category 4 + 5^b^, High is category 6 + 7^b^

^c^SMSSE, Standardized Mini Mental State Examination

^d^GDS, Geriatric Depression Scale

*p < 0.001; ^2^pearson chi square test;^1^t–test

SMMSE data were collected from 43 residents in the small scaled group (intervention) and from 41 residents in the regular care group (control). Information about mood (GDS) was assessed in 37 residents in the intervention group and in 31 residents in the control group. Observational data was collected for 68 residents in the intervention group and 52 residents in the control group at baseline.

The QOL variable ‘suspicious behavior’ shows a significant difference at baseline (*p* = .017) implicating more suspicious behavior in the regular care group (t-test, equal variances not assumed, *p* > 0.05). For all other QOL variables no significant differences were found at baseline (see Table 3). For both groups, at baseline, residents show average [38] scores on the QUALIDEM items care relationship, positive and negative affect, restless behavior, positive self-image, social isolation, feeling at home and having something to do. A score below (1 SD) average was found for having social relations. The GIP scores show scores slightly above average (1/2–1 *SD*) ([33, 39]) for not social behavior and apathy. The subscales insubordinate, suspicious, depressive and anxious behavior showed average scores. Overall, the mean scores between the three measurements of both groups showed no significant differences.

**Table 3:** Raw scores, *M, SD*

Small scaled homelike group (*n* = 54)							Control group (*n* = 30)						
T1		T2		T3			T1		T2		T3		
	M *(SD)*	95% CI	M *(SD)*	95% CI	M *(SD)*	95% CI	M *(SD)*	95% CI	M *(SD)*	95% CI	M *(SD)*	95%CI	Range
QUALIDEM													
Care relationship	13.4 *(3.6)*	12.4–14.4	13.9 *(4.1)*	12.7–15.0	13.3 *(4.4)*	12.1–14.4	14.8 *(3.6)*	13.5–16.1	14.1 *(4.5)*	12.5–15.6	14.9 *(4.1)*	13.3–16.5	0–21
Positive affect	12.2 *(3.8)*	11.1–13.2	12.2 *(3.6)*	11.2–13.2	11.8 *(4.0)*	10.6–13.0	12.2 *(3.9)*	10.8–13.6	12.2 *(4.7)*	10.7–13.6	11.7 *(4.9)*	10.1–13.3	0–18
Negative affect	5.5 *(2.2)*	4.9–6.1	5.5 *(1.9)*	5.0–6.0	6.0 *(2.1)*	5.5–6.6	5.7 *(2.2)*	4.9–6.5	6.2 *(2.1)*	5.5–7.0	5.5 *(2.4)*	4.7–6.3	0–9
Restless tense behavior	4.7 *((2.7)*	4.0–5.4	4.3 *(2.5)*	3.7–5.0	4.3 *(2.5)*	3.6–5.0	4.1 *(2.6)*	3.2–5.1	4.4 *(2.5)*	3.5–5.3	3.9 *(2.7)*	3.0–4.9	0–9
Positive self image	6.6 *(2.5)*	6.0–7.3	6.7 *(2.6)*	6.07.4	6.7 *(2.8)*	6.0–7.5	6.9 *(2.4)*	6.1–7.8	7.5 *(2.3)*	6.6–8.4	6.9 *(2.7)*	5.9–7.9	0–9
Social relations	9.7	8.6–10.7	9.1	8.1–10.2	8.6	7.5–9.6	8.7	7.3–10.1	8.3	6.8–9.7	8.5	7.1–9.9	0–18
Social isolation	5.9	5.3–6.5	5.4	4.8–6.1	5.9	5.2–6.6	6.3	5.5–7.1	6.5	5.7–7.4	5.9	5.0–6.8	0–9
Feeling at home	9.6	8.9–10.2	9.6	8.9–10.2	9.5	8.7–10.3	9.5	8.6–10.3	10.4	9.5–11.6	10.6	9.5–11.6	0–12
Having something to do	1.6	1.1–2.1	1.5	1.1–2.0	1.2	0.7–1.6	1.9	1.2–2.5	1.3	0.7–1.9	1.1	0.5–1.7	0–6
GDS-15													
	1.3	0.8–1.7	1.1	0.7–1.6	0.8	0.5–1.2	1.0	0.5–1.5	1.0	0.5–1.5	0.8	0.4–1.2	0–15
GIP													
Not social behavior	20.5	19.1–21.8	20.9	19.5–22.3	21.1	19.8–22.5	19.5	17.6–21.3	21.1	19.2–12.0	20.6	18.7–22.4	8–32
Apathy	15.0	14.0–16.1	15.3	14.2–16.4	15.3	14.2–16.3	14.6	13.2–16.0	15.9	13.2–16.0	15.4	14.0–16.9	6–24
Insubordinate behavior	10.1	9.3–11.0	10.0	9.3–10.7	9.8	9.0–10.6	10.2	9.1–11.3	10.6	9.6–11.5	10.0	9.0–11.0	5–20
Suspicious behavior	9.9	8.5–11.2	9.6	8.4–10.9	8.7	7.6–9.9	11.3	9.5–13.0	10.7	9.1–12.4	9.5	8.0–11.0	7–28
Depressive behavior	10.0	9.0–10.9	9.7	8.7–10.7	9.3	8.4–10.2	9.6	8.3–10.8	9.2	7.9–10.6	9.1	7.9–10.3	6–24
Anxious behavior	8.1	7.2–9.2	8.3	7.3–9.4	7.9	7.0–8.8	9.8	8.5–11.1	10.3	8.9–11.8	9.7	8.4–10.9	6–24

### Differences in quality of life aspects over time

The raw subscale scores of the 9 QUALIDEM subscale scores, the GDS-15 scores and the 6 GIP scores are presented in Table 3. A significant difference over time between both groups was found for the GIP subscale anxious behavior. Residents in the small-scaled homelike group show significant less anxious behavior compared to the regular care group, with a moderate effect size. The difference in raw score of the small-scaled homelike group (7.9) and the regular care group (9.7) was 1.8 points (follow up measure). No significant differences were found for the other variables (Table 4).

**Table 4:** *p*-values and eta square quality of life subscales both conditions

	*P* value^a^	*P* value^b^	Eta square
QUALIDEM			
Care relationship	.960^d^	.158	.024
Positive affect	.789^c^	.955	.000
Negative affect	.610^c^	.813	.001
Restles tense behavior	.756^c^	.541	.005
Positive self image	.313^c^	.377	.010
Social relations	171^c^	.415	.008
Social isolation	.615^c^	.273	.015
Feeling at home	.748^c^	.235	.017
Having something to do	.678^c^	.951	.000
GDS			
Mood	.187^c^	.509	.013
GIP			
Not social behavior	.867^c^	.650	.003
Apathy	.896^c^	.902	.000
Insubordinate behavior	.558^c^	.617	.003
Suspicious behavior	.017^d^*	.222	.019
Depressive behavior	.786^c^	.635	.003
Anxious behavior	.072^d^	.008*	.086

^a^at baseline

^b^time x group

^c^equal variances assumed

^d^equal variances not assumed

*significant

## Discussion

In the present study, we compared QOL of residents with dementia living in a small scaled homelike SCU with that of residents living in a regular large-scale SCU. Symptoms of depression and agitation are related to a lower QOL [40]. In our study, residents in a small scaled homelike SCU show significant less anxious behavior compared to residents in a regular SCU over time. Because of the high prevalence of anxiety and relation with other neuropsychiatric symptoms among persons with dementia in nursing homes [41–43], we believe this finding is of clinical relevance. QOL, is associated with the presence of neuropsychiatric symptoms [40, 44].

No other significant differences were found between both groups on other aspects of quality of life. Other research however, showed differences in quality of life, measured by the QUALIDEM, between traditional nursing homes and green care farms [45]. Residents in traditional nursing homes scored lower on positive affect, social relations and having something to do, compared with green care farms, with no differences in quality of life between small scaled living facilities and green care farms [45]. Research on green houses and traditional nursing homes also show more QOL in favor of the small scaled facility [46].

One explanation for these differences is that most of our participants had relatively high scores on the QUALIDEM items. This may implicate that quality of life and wellbeing was already substantially high in both groups at the outline of the study and further improvement of QOL may not have been possible to register with the QUALIDEM due to ceiling effects [47]. Only the item ‘having something to do’ showed low baseline scores and this score remained low. In addition, the scores on the GIP questionnaire were also relatively high, implicating the presence of substantial neuropsychiatric behavior in our resident groups. It is possible that this explains why these behaviors responded to our intervention. Our data showed that moving to a small scaled SCU was associated with a decrease of anxiety in our resident group. Anxiety is reported to be present in one third of the residents living in a nursing home [48] and is therefore a very common neuropsychiatric symptom that also is related to wellbeing and quality of life in residents with dementia [49]. The specific decrease of anxiety in small scaled homelike SCUs may be related to the more homelike aspects of these units, including more social support, safety and a recognizable familiar environment [19]. It is possible that the type of care did not have an effect on depression and quality of life [10, 50, 51] since our data indicate that both resident groups had already high levels of well-being before entering the study (as estimated by GDS and QOL measures).

In addition, it could be argued that the lack of effects on QOL was due to confounding effects of other factors such as, for example, the use of psychotropic drugs. So far, we have neither data on medication used at the time of testing, nor do we know whether the groups differed in the type or amount of drugs prescribed. Furthermore we chose to measure QOL with observations by nursing staff because we believed this to be more accurate than interviews with the residents themselves [16, 52]. It is possible that additional observations by the resident’s spouses or other relatives would have improved the sensitivity of our measurements. These are issues that should be worthwhile to pursue in subsequent research.

### Strengths and weaknesses of the study

One major strength of our study is its longitudinal design. The type of research that has been done in this field used mostly cross-sectional designs [15]. The advantage of using within-subject measurements is that potential confounding effects of individual differences are minimized. A second strength is that we used the QUALIDEM, an assessment tool that is widely used in other studies as well [52] and is well known for its strong methodological features [8, 53]. The other assessment tool (GIP) however is only generally used in the Netherlands. In future research the use of other instruments as, for example, the Greater Cincinnati Chapter Well- Being Observation Tool or the Psychosocial Quality of Life Domains questionnaire should be considered [32, 54]. Finally, another merit of our study is that we included a relatively large sample of residents with dementia.

A potential limitation of the present study is the lack of randomization. We did not allocate residents randomly to one of the experimental conditions because of ethical motives in that residents and families should be free to choose the living environment they prefer. Non-randomization can have caused a bias in the answers of the nurses because they expected a better care environment for the residents after the relocation. Another drawback may be that our nurses who assessed the data were not blind to the experimental condition of the resident. This could have influenced the results. Nevertheless, we believe that the within subject design we used forms a major strength of this trial, together with the fact that our resident groups did not differ on quality of life at baseline.

Because of the severely cognitive impaired population, direct assessment measurements with the residents was hardly possible. We collected convergent information about mood by assessing the Geriatric Depression Scale questionnaire verbally to the residents to increase the possibility of a valid answer. However, the answers of the residents will stay limitedly valid.

The QUALIDEM domains positive ‘self-image’, ‘feeling at home’ and ‘having something to do’ cannot be assessed by residents with very severe dementia [55]. In our research, Global cognitive functioning was assessed with the SMSSE. Considering the very low SMSSE scores, we assume that the data presented for these specific subscales is of very limited value. In further research, the stages of dementia severity could be classified conform the Global Deterioration Scale and Functional Assessment Staging [56]. It is relevant to include residents with very severe dementia in future studies.

A final weakness of this research is the lack of registration of comorbidities of the residents besides the type of dementia and neuropsychiatric symptoms. Including comorbid diseases would be preferable in future research.

## Conclusions

The study demonstrates that moving to a small-scale care facility is associated with a reduction of anxiety in residents with dementia. These findings add to growing evidence supporting the benefits of small, homelike care facilities on well-being of these residents. The experience of decreased anxiety in residents in small scaled homelike facilities was clinically relevant. One aim of further research could be to unravel what specific aspects of small-scaled living environments cause this reduction in anxiety in dementia. For example, this may be related to the type of psychosocial climate, certain physical aspects of the environment, the decreased burden in family member or perhaps the increased work satisfaction of the nursing staff. Researching variation and diversity in physical an psychosocial climate within nursing facilities in relation to the former and current individual characteristics of residents can perhaps contribute to a better understanding of the effective aspects of dementia care.

## Abbreviations

CI: Confidence Interval
GDS: Geriatric Depression Scale
GIP: Gedragsobservatieschaal voor Intramurale Psychogeriatrie
MMSE: Mini Mental State Examination
QOL: Quality of Life
SCU: Special Care Unit

## References

[1] Moyle W, Fetherstonhaugh D, Greben M, Beattie E (2015). Influencers on quality of life as reported by people living with dementia in long-term care: a descriptive exploratory approach. BMC Geriatr..

[2] Crellin NE, Orrell M, McDermott O, Charlesworth G (2014). Self-efficacy and health-related quality of life in family carers of people with dementia: a systematic review. Aging Ment Health..

[3] Clare L, Quinn C, Hoare Z, Whitaker R, Woods RT (2014). Care staff and family member perspectives on quality of life in people with very severe dementia in long-term care: a cross-sectional study. Health Qual Life Outcomes..

[4] Bentvelzen A, Aerts L, Seeher K, Wesson J, Brodaty H. A Comprehensive Review of the Quality and Feasibility of Dementia Assessment Measures: The Dementia Outcomes Measurement Suite. J Am Med Dir Assoc. 2017; 10.1016/j.jamda.2017.01.006. 10.1016/j.jamda.2017.01.006 28283381

[5] Bravo G, Sene M, Arcand M (2017). Reliability of health-related quality-of-life assessments made by older adults and significant others for health states of increasing cognitive impairment. Health Qual Life Outcomes..

[6] Gräske J, Fischer T, Kuhlmey A, Wolf-Ostermann K (2012). Quality of life in dementia care--differences in quality of life measurements performed by residents with dementia and by nursing staff. Aging Ment Health..

[7] Black BS, Johnston D, Morrison A, Rabins PV, Lyketsos CG, Samus QM (2012). Quality of life of community-residing persons with dementia based on self-rated and caregiver-rated measures. Qual Life Res..

[8] Page TE, Farina N, Brown A, Daley S, Bowling A, Basset T, Livingston G, Knapp M, Murray J, Banerjee S (2017). Instruments measuring the disease-specific quality of life of family carers of people with neurodegenerative diseases: a systematic review. BMJ Open..

[9] de Rooij AH, Luijkx KG, Declercq AG, Schols JM (2011). Quality of life of residents with dementia in long-term care settings in the Netherlands and Belgium: design of a longitudinal comparative study in traditional nursing homes and small-scale living facilities. BMC Geriatr..

[10] van Kooten J, van der Wouden JC, SAM S, Smalbrugge M, CMPM H, Stek ML. Pain, Neuropsychiatric Symptoms, and Quality of Life of Nursing Home Residents With Advanced Dementia in The Netherlands: A Cross-sectional Study. Alzheimer Dis Assoc Disord. 2017; 10.1097/WAD.0000000000000197. 10.1097/WAD.0000000000000197 28486239

[11] Baquero M, Martín N (2015). Depressive symptoms in neurodegenerative diseases. World J Clin Cases..

[12] Almeida OP, MacLeod C, Flicker L, Ford A, Grafton B, Etherton-Beer C (2014). RAndomised controlled trial to imProve depressIon and the quality of life of people with Dementia using cognitive bias modification: RAPID study protocol. BMJ Open..

[13] Mjorud M, Kirkevold M, Rosvik J, Selbaek G, Engedal K (2014). Variables associated to quality of life among nursing home patients with dementia. Aging & Mental Health..

[14] Abrahamson K, Lewis T, Perkins A, Clark D, Nazir A, Arling G (2013). The influence of cognitive impairment, special care unit placement, and nursing facility characteristics on resident quality of life. J Aging Health..

[15] Kok JS, Berg IJ, Scherder EJ (2013). Special care units and traditional care in dementia: relationship with behavior, cognition, functional status and quality of life - a review. Dement Geriatr Cogn Dis Extra..

[16] Crespo M, Hornillos C, de Quirós MB (2013). Factors associated with quality of life in dementia patients in long-term care. Int Psychogeriatr..

[17] Cadigan RO, Grabowski DC, Givens JL, Mitchell SL (2012). The quality of advanced dementia care in the nursing home: the role of special care units. Med Care..

[18] Verbeek H, Zwakhalen SM, van Rossum E, Ambergen T, Kempen GI, Hamers JP (2010). Small-scale, homelike facilities versus regular psychogeriatric nursing home wards: a cross-sectional study into residents' characteristics. BMC Health Serv Res..

[19] Kim SK, Park M (2017). Effectiveness of person-centered care on people with dementia: a systematic review and meta-analysis. Clin Interv Aging..

[20] Verbeek H, van Rossum E, Zwakhalen SM, Kempen GI, Hamers JP (2009). Small, homelike care environments for older people with dementia: a literature review. Int Psychogeriatr..

[21] Pot AM, de Lange J (2010). Monitor woonvormen Dementie; een studie naar verpleeghuiszorg voor mensen met dementie. Trimbos Instituut Netherlands Institute of Mental Health and Addiction.

[22] Verbeek H, Zwakhalen SM, van Rossum E, Ambergen T, Kempen GI, Hamers JP (2010). Dementia care redesigned: Effects of small-scale living facilities on residents, their family caregivers, and staff. J Am Med Dir Assoc..

[23] Fleming R, Kelly F, Stillfried G (2015). ‘I want to feel at home’: establishing what aspects of environmental design are important to people with dementia nearing the end of life. BMC Palliat Care..

[24] Nikmat AW, Hawthorne G, Al-Mashoor SH (2015). The comparison of quality of life among people with mild dementia in nursing home and home care--a preliminary report. Dementia (London).

[25] Love K, Femia E (2015). Helping Individuals With Dementia Live More Fully Through Person-Centered Practices. J Gerontol Nurs..

[26] Folstein M, Folstein S, McHugh P (1975). Mini-Mental State: A practical method for grading the cognitive state of patients for de clinician. J Psychiatr Res.

[27] Kok R, Heeren T, Hemert van A (1993). De Geriatric Depression Scale. Tijdschrift voor psychiatrie..

[28] Yesavage J, Brink T, Rose T, Lum O, Huang V, Adey M, Leirer V (1982). Development and validation of a geriatric depression screening scale: a preliminary report. Journal of Psychiatric Research.

[29] Marc LG, Raue PJ, Bruce ML (2008). Screening performance of the 15-item geriatric depression scale in a diverse elderly home care population. Am J Geriatr Psychiatry..

[30] Ettema TP, Dröes RM, de Lange J, Mellenbergh GJ, Ribbe MW (2007). QUALIDEM: development and evaluation of a dementia specific quality of life instrument--validation. Int J Geriatr Psychiatry..

[31] Ettema TP, Dröes RM, de Lange J, Mellenbergh GJ, Ribbe MW (2007). QUALIDEM: development and evaluation of a dementia specific quality of life instrument. Scalability, reliability and internal structure. Int J Geriatr Psychiatry..

[32] Aspden T, Bradshaw SA, Playford ED, Riazi A (2014). Quality-of-life measures for use within care homes: a systematic review of their measurement properties. Age Ageing..

[33] Verstraten P, van Eekelen C (1987). Handleiding voor de Gedragsobservatieschaal voor intramurale psychogeriatrie. Van Loghum Slaterus.

[34] Robey R (2004). Reporting point and interval estimates of effect-size for planned contrasts: fixed within effect analyses of variance. J Fluency Disord.

[35] Kok JS, van Heuvelen MJ, Berg IJ, Scherder EJ (2016). Small scale homelike special care units and traditional special care units: effects on cognition in dementia; a longitudinal controlled intervention study. BMC Geriatr..

[36] Kok JS, Berg IJ, Blankevoort CG, Scherder EJA. Rest-activity Rhythms in Small Scale Homelike Care and Traditional Care for Residents with Dementia. 2017 June. BMC Geriatr. 2017; 10.1186/s12877-017-0525-1. 10.1186/s12877-017-0525-1 PMC5498984 28679366

[37] Verhage F (1964). Intelligence and Age: Research on Dutch people aged twelve to seventy seven years old.

[38] Graske J, Meyer S, Wolf-Osterman K (2014). Quality of life ratings in dementia care: a cross-sectional study to identify factors associated with proxy-ratings. Health Qual Life Outcomes..

[39] Normative data for neuropsychological tests. http://www.normgroepen.nl/Vragenlijst/GDS-15-pg-vph/. Accessed 3 Nov 2017.

[40] Beerens HC, Zwakhalen SMG, Verbeek H, Ruwaard D, Hamers JPH (2013). Factors associated with quality of life of people with dementia in long-term care facilities: a systematic review. Int J Nurs Stud..

[41] Goyal AR, Bergh S, Engedal K, Kirkevold M, Kirkevold O (2017). Anxiety, Anxiety Symptoms, and Their Correlates in Persons with Dementia in Norwegian Nursing Homes: A Cause for Concern. Dement Geriatr Cogn Disord..

[42] Jao YL, Loken E, MacAndrew M, Van Haitsma K, Kolanowski A. Association between social interaction and affect in nursing home residents with dementia. Aging Ment Health. 2017:1–6. 10.1080/13607863.2017.1304526. 10.1080/13607863.2017.1304526 28332405

[43] Bidzan M, Bidzan L, Pachalska M (2014). Neuropsychiatric symptoms in patients with Alzheimer's disease with a vascular component. Ann Agric Environ Med..

[44] Mjørud M, Røsvik J, Rokstad AM, Kirkevold M, Engedal K (2014). Variables associated with change in quality of life among persons with dementia in nursing homes: a 10 months follow-up study. PLoS One..

[45] de Boer B, Hamers JP, Zwakhalen SM, Tan FE, Beerens HC, Verbeek H (2017). Green Care Farms as Innovative Nursing Homes, Promoting Activities and Social Interaction for People With Dementia. J Am Med Dir Assoc..

[46] Kane RA, Lum TY, Cutler LJ, Degenholtz HB, Yu TC (2007). Resident outcomes in small-house nursing homes: a longitudinal evaluation of the initial green house program. J Am Geriatr Soc..

[47] Dichter MN, Schwab CG, Meyer G, Bartholomeyczik S, Halek M (2016). Item distribution, internal consistency and inter-rater reliability of the German version of the QUALIDEM for people with mild to severe and very severe dementia. BMC Geriatr..

[48] Goyal AR, Bergh S, Engedal K, Kirkevold M, Kirkevold Ø (2017). Anxiety, Anxiety Symptoms, and Their Correlates in Persons with Dementia in Norwegian Nursing Homes: A Cause for Concern. Dement Geriatr Cogn Disord..

[49] Haaksma ML, Leoutsakos JS, Bremer JA, Aalten P, Ramakers IH, Verhey FR, Olde Rikkert MG, Melis RJ. The clinical course and interrelations of dementia related symptoms. Int Psychogeriatr. 2017:13:1–8. 10.1017/S1041610217000321. 10.1017/S1041610217000321 28285610

[50] Hongisto K, Hallikainen I, Selander T, Törmälehto S, Väätäinen S, Martikainen J, Välimäki T, Hartikainen S, Suhonen J, Koivisto AM. Quality of Life in relation to neuropsychiatric symptoms in Alzheimer's disease: 5-year prospective ALSOVA cohort study. Int J Geriatr Psychiatry. 2017; 10.1002/gps.4666. 10.1002/gps.4666 28067961

[51] Pusswald G, Moser D, Pflüger M, Gleiss A, Auff E, Stögmann E, Dal-Bianco P, Lehrner J (2016). The impact of depressive symptoms on health-related quality of life in patients with subjective cognitive decline, mild cognitive impairment, and Alzheimer's disease. Int Psychogeriatr..

[52] Oudman E, Veurink B. Quality of life in nursing home residents with advanced dementia: a 2-year follow-up. Psychogeriatrics. 2014;14(4):235–40. 10.1111/psyg.12062 25495085

[53] Klapwijk MS, Caljouw MA, Pieper MJ, van der Steen JT, Achterberg WP (2016). Characteristics Associated with Quality of Life in Long-Term Care Residents with Dementia: A Cross-Sectional Study. Dement Geriatr Cogn Disord..

[54] Algar K, Woods RT, Windle G (2016). Measuring the quality of life and well-being of people with dementia: A review of observational measures. Dementia (London)..

[55] Dichter MN, Dortmann O, Halek M, Meyer G, Holle D, Nordheim J, Bartholomeyczik S (2013). Scalability and internal consistency of the German version of the dementia-specific quality of life instrument QUALIDEM in nursing homes - a secondary data analysis. Health Qual Life Outcomes..

[56] Reisberg B (2007). Global measures: utility in defining and measuring treatment response in dementia. Int Psychogeriatr..

